# The Relationship Between Neuroticism Fit and General Well-Being: The Mediating Effect of Psychological Resilience

**DOI:** 10.3389/fpsyg.2019.02219

**Published:** 2019-10-04

**Authors:** Ran Hao, Huan Dong, Ruili Zhang, Ping Li, Peng Zhang, Meng Zhang, Jie Hu

**Affiliations:** ^1^Department of Clinical Humanistic Care and Nursing Research Center, School of Nursing, Hebei Medical University, Shijiazhuang, China; ^2^Aerospace Center Hospital, Beijing, China

**Keywords:** neuroticism, older adults, primary caregivers, congruence, psychological resilience

## Abstract

**Background:**

The dominance complementarity theory argues that effective and continuing interpersonal relationships require complementary dominance and submission values. This theory has been widely applied to interpersonal interaction studies. Although studies have demonstrated the correlation between neurotic personality traits and general well-being (GWB) in older adults, the interpersonal interactions and psychological mechanisms underlying this effect remain unclear.

**Aim:**

Using this theory, we explored the effect of the neuroticism fit between older adults and primary caregivers on older adults’ GWB and examined the mediating role of psychological resilience (PR).

**Methods:**

One hundred sixty-one dyads of older adults and primary caregivers in nursing homes completed scales that included the Eysenck Personality Questionnaire-Revised Short Scale, the 10-Item Connor-Davidson Resilience Scale, and the GWB Schedule. We performed a cross-level polynomial regression, response surface modeling and mediating effect test to analyze the data.

**Results:**

(1) Older adults’ GWB was higher when the neuroticism fit between older adults and primary caregivers was incongruent rather than congruent (*p* < 0.01). (2) In cases of incongruence, older adults’ GWB was higher only if their neuroticism was lower than that of their primary caregivers (*p* < 0.01). (3) In cases of congruence, older adults’ GWB was higher when the neuroticism of both sides was lower (*p* < 0.01). (4) PR partially mediated the relationship between neuroticism incongruence and older adults’ GWB (indirect effect = 0.14, *p* < 0.01).

**Conclusion:**

The neuroticism incongruence between older adults and primary caregivers was beneficial to older adults’ GWB and was partially mediated by PR.

## Introduction

Recently, the aging population has impacted the service quality of older adults in China, and more attention has been paid to mental health care for older adults. General well-being (GWB) refers to an individual’s overall assessment of the quality of life based on the individual’s own standard and is an important index for measuring the level of mental health (Fazio, 1977). GWB involves an emotional component (positive and negative emotions) and a cognitive component of life satisfaction (Diener et al., 2013). A high level of GWB is related to better health and lower morbidity (Diener and Chan, 2011). However, recent studies have demonstrated that in older adults, GWB appears to exhibit an age-related decline (Smith et al., 2002; Momtaz et al., 2011). It is not unusual for older adults in nursing homes to experience a low level of GWB (Timmins et al., 2015).

The factors influencing GWB can be divided into internal factors and external factors (Friedman and Kern, 2014). Personality, as an important internal factor, affects people’s psychological status and behavior patterns (Friedman and Kern, 2014). Neuroticism, which is one of the most important personality traits, is defined as emotional instability (Ormel et al., 2012). People with low neuroticism tend to be more emotionally stable and less reactive to stress. They tend to be calm, even tempered, and less likely to feel tense or rattled. Although they are low in negative emotion, they are not necessarily high in positive emotion (DeNeve and Cooper, 1998). In contrast, people high in neuroticism are more likely to be moody, to have worse responses to stressors, and to experience feelings such as anxiety, worry, fear, anger, frustration, guilt, depression, and loneliness (Thompson, 2008).

With regard to GWB in older adults, current studies have mainly focused on the characteristics of individuals’ own neuroticism. Most studies have shown that high levels of neuroticism predict low GWB for older adults (Custers et al., 2010; Friedman et al., 2010). Although researchers have indicated that neuroticism is a strong predictor of GWB from a personal perspective (Ozer and Benet-Martinez, 2006; Friedman et al., 2010), one critical question involves the effect of interpersonal interaction on older adults’ GWB.

Older adults in nursing homes generally do not live alone. On a daily basis, they obtain the majority of their physical and psychological support from their primary caregivers. These primary caregivers might play a critical role in affecting older adults’ GWB. Research has shown that caregivers’ personality traits are important determinants of care recipients’ well-being, which are moderated by relationship satisfaction (Ruiz et al., 2006). Higher neuroticism of caregivers predicted higher care recipients’ depressive symptoms in a follow-up study (Ruiz et al., 2006). As Abe and Oshio (2018) shown that, incongruence neuroticism between individuals contributes to maintaining positive interactions and generating high satisfaction. However, previous studies overlooked the neuroticism interaction between individuals. Since primary caregivers’ neuroticism play a critical role in affecting older adults’ well-being (Ruiz et al., 2006), we propose that the latter’s neuroticism might influence the former’s emotions and behaviors in turn, and the neuroticism interaction between two parties might affect the latter’s GWB. That is to say, older adults’ GWB might be affected not only by the neuroticism of older adults and primary caregivers alone, but also by the neuroticism interaction of the dyads (i.e., older adult and his/her primary caregiver). Thus, it is important to examine the neuroticism fit between dyads of older adults and their primary caregivers rather than to examine only the former’s or the latter’s neuroticism in predicting the former’s GWB.

The dominance complementarity theory (Carson, 1969; Kiesler, 1983) has been widely applied to interpersonal interaction studies. Prior research has verified that roommate dyads with dissimilar neuroticism tend to produce more positive emotion and behavioral attraction compared to those with similar neuroticism (Hudson and Fraley, 2014). Applications of this theory have also identified a positive correlation between extraversion personality incongruence of leader-follower dyads and follower job engagement, and it were based on an increased balance of job resources and reduced conflicts (Chen et al., 2016). For older adults in nursing homes, primary caregivers are their key interactive personal environment. Thus, older adults’ GWB might be affected by the neuroticism fit between themselves and their primary caregivers.

In addition, the neuroticism influences corresponding psychological processes, such as cognition and emotion, which further influence psychological resilience (PR) and older adults’ GWB in turn (Campbell-Sills et al., 2006). PR refers to the ability to adapt to changing situations and recover from negative emotions. It is affected by the external environment and creates a dynamic balance (Block and Kremen, 1996). He et al. (2013) contended that PR partially mediated the relationship between dispositional optimism and well-being, when dispositional optimism acted as a protective factor, by increasing the ability of an individual to recover from frustrations. Moreover, higher PR predicted greater happiness, lower depression, and greater satisfaction with life in older adults (i.e., greater psychological well-being) (Tomás et al., 2012; Smith and Hollingersmith, 2015). Additionally, a negative association with neuroticism in young adults was reported (Wiltermuth et al., 2015). In the United States, a high PR group of emerging adults and young adults showed lower neuroticism than a low PR group (Shiner and Masten, 2012). Previous studies mainly investigated the relationship among GWB, neuroticism and PR in personal perspective, instead of interpersonal perspective. Particularly, we wondered the PR-based mechanism that links neuroticism fit to older adults’ GWB. In this study, based on dominance complementarity theory, we aimed to explore the effect of neuroticism fit between older adults and their primary caregivers on older adults’ GWB and to further examine the mediating role of PR.

## Theoretical Background and Hypotheses Development

### Dominance Complementarity Theory

Dominance complementarity theory argues that effective and continuing interpersonal relationships require complementary dominance and submission values (Carson, 1969; Kiesler, 1983); that is, one party assumes a dominant and controlling role while the other party assumes a submissive and docile role, and both parties agree with these roles (Carson, 1969). Specifically, dyads with dominance complementarity can reduce uncertainty, conflict and competition and promote achievement in a work situation (Tiedens et al., 2007). However, a lack of dominance complementarity causes power struggles, disharmony and dissatisfaction (Ross et al., 2016). Therefore, in nursing homes, primary caregivers develop unique dyadic relationships with each individual older adult, and the quality of these relationships ranges from low to high.

### Neuroticism Congruence/Incongruence and Older Adults’ GWB

According to dominance complementarity theory, high-quality interactions are facilitated when dominance and assertiveness from one party are balanced by compliance, obedience, and submissiveness from the other party (Carson, 1969; Kiesler, 1983). People with higher neuroticism are prone to experience more negative emotions and to have less self-confidence (Judge et al., 1997), which would make them doubt whether they can successfully bring about changes (Morrison and Phelps, 1999). Research demonstrates that neuroticism is theoretically considered to be negatively correlated with proactive behavior. As Côté and Moskowitz (1998) suggested, people high in neuroticism will engage in less proactive and dominant behavior in social interactions to avoid less pleasant affect. There are strong evidence that the trait of neuroticism is related to the interpersonal circumplex trait of submissiveness and to questionnaire reports of submissive behavior (Trapnell and Wiggins, 1990; Gilbert and Allen, 1994). From this perspective, older adults with high neuroticism tend to complement the calm, steady behaviors from primary caregivers with low neuroticism, but to conflict with primary caregivers with high neuroticism.

Prior research has indicated that the incongruence of neuroticism between individuals contributes to maintaining positive interactions and generating high satisfaction (Abe and Oshio, 2018). Luther and Benkenstein (2017) found that incongruence in neuroticism was beneficial for roommates, and more positive emotions and behavior presented in dissimilar neurotic roommates than in similar ones. We assume that dyadic incongruence in neuroticism may serve as the basis for mutually emotional communication that produces a high-quality relationship and contributes to older adults’ GWB. A critical role for primary caregivers is to take care of older adults, therefore, the former’s’ emotional adjustment status and job performance may affect the latter’s satisfaction in dyadic relationships and GWB. In contrast, congruence in neuroticism is likely to be detrimental to dyadic relationships because congruence in neuroticism may prevent the dyadic members from forming more positive emotions, and make it difficult to develop high-quality relationships (Luther and Benkenstein, 2017). Thus, this condition may promote older adults’ GWB. Therefore, we hypothesize the following:

Hypothesis 1. Older adults’ GWB is higher when the neuroticism fit between older adults and their primary caregivers is incongruent rather than congruent.

It is also important to differentiate two scenarios of incongruence in neuroticism, i.e., high-low and low-high (older adults-primary caregivers). When older adults’ neuroticism is lower than that of their primary caregivers, the former can better control emotions and handle stressful events when they are in dominant positions (Trapnell and Wiggins, 1990; Gilbert and Allen, 1994). This assists primary caregivers in reducing negative emotions, facilitates the dyadic emotional communication, and results in an increase in the GWB of older adults. Moreover, primary caregivers who are high in neuroticism are predisposed to show more detailed care and higher execution, which are the most important qualities to their work (Perkins et al., 2015). In contrast, when older adults’ neuroticism is higher than that of their primary caregivers, older adults in obedient positions are emotional and anxious (Otonari et al., 2012), whereas their primary caregivers are emotionally stable, and insufficiently sensitive. In this situation, it is difficult for older adults to be satisfied with the work of their primary caregivers, which reduces older adults’ emotional communication and GWB. Even if they can obtain support and help from their primary caregivers, the older adults’ GWB would be lower than in the former case. Thus, we assume the following:

Hypothesis 2. In cases of incongruence, older adults’ GWB is higher when their neuroticism is lower than that of primary caregivers rather than vice versa.

Specifically, when high neuroticism congruence exists between older adults and their primary caregivers, both parties are emotional and irritable (Otonari et al., 2012), which tends to produce more negative emotions and vicious circles, and to worsen older adults’ mental health and their GWB. However, when the neuroticism between the dyads of older adults and primary caregivers is congruent at low levels, they are both in positions of emotional control (Ozer and Benet-Martinez, 2006). As a result, there will be more positive emotions and behaviors. Thus, we hypothesize the following:

Hypothesis 3. In cases of congruence, older adults’ GWB is higher when older adults and primary caregivers are aligned at a low level of neuroticism rather than aligned at a high level.

### PR as a Mediator of the Incongruence Effect on GWB

Prior research has established positive links between PR and well-being (Li et al., 2017). When individuals possess high PR, they are able to cope with stressful events and deal with negative emotions (Campbell-Sills et al., 2006), resulting in a high level of well-being. It has been demonstrated that PR was negatively correlated with neuroticism (Morales et al., 2018). There is evidence that people with high neuroticism possess more vulnerable emotions and poor coping styles (Ormel et al., 2012). In contrast, people low in neuroticism are characterized by emotional stability and the ability to cope with stress (Ormel et al., 2012). Given that we have hypothesized an incongruence effect in neuroticism (i.e., Hypothesis 1) and given the established relationship among PR, neuroticism and GWB, we expect a mediating effect of PR between neuroticism incongruence and GWB.

Hypothesis 4. Increased incongruence between older adults and primary caregivers would predicted higher older adults’ GWB partially mediated by older adults’ PR.

## Materials and Methods

### Subjects

The participants were enrolled from 18 nursing homes in Shijiazhuang City, located in northern China. We investigated 194 dyads of older adults and primary caregivers, and the response rate for the questionnaires was 82.98%. Ultimately, questionnaires from 161 dyads were included in this study. The inclusion criteria for older adults were (1) age over 60 years, (2) clear consciousness and stable symptoms, and (3) informed consent. The exclusion criteria were (1) abnormal language communication, (2) auditory dysfunction, and (3) severe psychiatric history and cognitive disorder. The above criteria were also applied to primary caregivers, with the exception of age. In addition, the primary caregivers had been continuously taking care of older adults for at least 1 month. In nursing homes, each older adult is paired with only one primary caregiver, but each primary caregiver might be paired with one or more older adults.

### Procedure

First, we contacted the managers of each nursing home and requested permission. If we received authorization, we began to recruit participants according to our criteria. To conduct the survey, we first distributed questionnaires to older adults and then to their primary caregivers, simultaneously ensuring their pairing. The first page of the questionnaire outlined the purpose of the study, its voluntary nature, and an assurance of confidentiality. This study was approved by the ethics committee of Hebei Medical University, and each participant provided verbal informed consent. All participants were instructed to return their completed questionnaires on the spot, with researchers assisting in explaining and filling out the questionnaires if necessary. Each participant received a gift for his/her participation.

### Measures

#### Eysenck Personality Questionnaire-Revised Short Scale for Chinese (EPQ-RSC)

We assessed older adults’ and primary caregivers’ personality using the 48-item EPQ-RSC, developed by Eysenck (Eysenck et al., 1985) and revised by Qian (Mingyi et al., 2000). The EPQ-RSC is applicable to Chinese adults aged 16 and over, including older adults. It consists of four subdimensions, including extraversion, neuroticism, psychoticism and a lie detector inventory, and each subdimension with 12 items, respectively. High scores of extraversion indicate extroversion, optimism and sociality. High scores of neuroticism indicate emotional instability characterized by depression, irritability, and anxiety. High scores of psychoticism indicate withdrawal, indifference and hostility to others. High scores of lie indicate a greater tendency to hide true information. These scores were changed into *T* scores according to Chinese norm. *T* scores (<43.3, 43.3–56.7, and >56.7) were used to assign psychoticism scores into weak, strong, and middle psychogenic types; extroversion scores into extroverted, introverted, and middle types; and neuroticism scores into stable, unstable, and middle mood types, respectively. In this study, the reliability coefficients of neuroticism subscale were 0.79 for older adults and 0.66 for primary caregivers.

#### 10-Item Connor-Davidson Resilience Scale (CD-RISC-10)

The Connor-Davidson Resilience Scale-10 was used to measure PR in older adults. The CD-RISC-10, developed by Connor and Davidson and modified by Campbell-Sills and Stein (2007), is often used to assess PR (Connor and Davidson, 2003). Each item is rated on a 5-point scale from 0 (not true at all) to 4 (true nearly all the time), and higher scores represent stronger PR. The reliability coefficient of the CD-RISC-10 was 0.88 for older adults.

#### General Well-Being Schedule (GWBS)

The 18-item GWBS was used to assess subjective GWB for older adults. The first 14 items use a six-point scale, and the others use a 0–10 scale (Fazio, 1977). The total possible scores range from 1 to 110, and higher scores represent better status (Fazio, 1977). Prior studies have indicated the satisfactory psychometric properties of the Chinese version of the GWBS (Duan, 1996; Tang, 2002). The present study found that the reliability coefficient of the GWBS was 0.80 for older adults.

#### Control Variables

A previous study suggested that GWB may be correlated with demographic variables such as gender and age (Zyphur et al., 2015). Thus, we introduced two control variables into our analysis: the gender and age of older adults and primary caregivers.

### Data Analysis

#### Confirmatory Factor Analysis

In the study, we examined the discriminant validity of the variables with confirmatory factor analysis (CFA) using Mplus (Mplus 7.0, Informer Technologies Inc., Los Angeles, CA, United States). Three factors were modeled as indicators of latent constructs (i.e., older adults’ neuroticism, older adults’ PR, and older adults’ GWB).

#### Cross-Level Polynomial Regressions and Response Surface Modeling

It is widely believed that the use of difference scores will lead to many methodological problems, including decreased reliability and validity, and pseudo-correlation (Johns, 1981). In order to avoid these problems, Edwards and Parry (1993) proposed an alternative method to test the relationship between the degree of congruence between two constructs and other constructs. This method established the polynomial regression equation and could evaluate the congruence effect. Meanwhile, it could avoid the above methodological problems caused by the use of difference scores, and could directly test the hypothetical content which is of great significance in the fit study (Edwards and Cable, 2009). This method was approved and tested by some scholars (Kalliath et al., 1999; Ostroff et al., 2005). The polynomial regression estimates the linear and non-linear relationship between the two independent variables and the dependent variable, and the linear relationship between the interaction term of the two independent variables and the dependent variable. The two independent variables constitute the congruence/incongruence effects. In this study, the two independent variables were older adults’ and primary caregivers’ neuroticism, respectively. And the dependent variable was older adults’ GWB. To examine the relationship between neuroticism congruence (i.e., high-high and low-low)/incongruence (i.e., high-low and low-high) and older adults’ GWB, we performed polynomial regressions. We tested hypotheses 1–3 using polynomial regression (Jansen and Kristof-Brown, 2005) combined with response surface modeling (Edwards and Parry, 1993). Specifically, older adults’ GWB was regressed on the control variables as well as five polynomial terms (older adults’ neuroticism, primary caregivers’ neuroticism, older adults’ neuroticism squared, primary caregivers’ neuroticism squared, and older adults’ neuroticism multiplied by primary caregivers’ neuroticism). In this study, since part of the older adults shared the same primary caregivers, it’s possible for shared variance to be detected in the primary caregivers’ neuroticism scores (Jansen and Kristof-Brown, 2005). The non-independence could bias the standard error estimates, leading wrong conclusion. The hierarchical linear modeling (HLM) allows multiple levels of variables in a nested structure to be formally represented by a submodel at its own level, and the submodel contributes to specifying how variables at one level affects other variables at another level. Thus, we incorporated our polynomial regression model within HLM (i.e., cross-level polynomial regression) (Jansen and Kristof-Brown, 2005). Older adults’ GWB and older adults’ neuroticism were analyzed at level 1, while primary caregivers’ neuroticism was analyzed at level 2. As shown in Eqs 1–4.

Level-1 Equation

(1) $$Y=\beta_{0}+\beta_{1}^{*}\,O+\beta_{2}^{*}\,O^{2}+e$$

Level-2 Equation

(2) $$\beta_{0}=\gamma_{\text{nn}}+\gamma_{\text{n}\,1}^{*}\,P+\gamma_{\text{n}\,2}^{*}\,P^{2}+u_{\text{n}}$$

(3) $$\beta_{1}=\gamma_{10}+\gamma_{11}^{*}\,P+u_{1}$$

(4) $$\beta_{2}=\gamma_{20}+u_{2}$$

Where Y represents the GWB of older adults, O is the neuroticism of older adults, and P is primary caregivers. To reduce multicollinearity and interpret the results, we centered O and P before calculating the square and interaction terms (Edwards and Parry, 1993; Edwards, 2002). Next, we plotted the three-dimensional response surface using unstandardized regression coefficients of the equation (Origin 9.1), where O and P are located on the horizontal axis and Y is located on the vertical axis.

Hypothesis 1 was workable based on the fact that the curvature along the incongruence line (a4 = γ_02_–γ_11_+γ_20_) was significant and positive. To support the asymmetric incongruence effect, we examined whether the slope of the incongruence line (a3 = γ_01_–γ_10_) was significant and positive (i.e., Hypothesis 2). In this study, the a3 was significant and positive, indicating older adults’ GWB was better when their neuroticism was lower than that of their primary caregivers. In addition, if the a3 was significant and negative, it indicated that the older adults’ GWB was better when their neuroticism was higher than that of their primary caregivers. We tested the congruence effect when the slope of the congruence line (a1 = γ_01_+γ_10_) was significant and negative (i.e., Hypothesis 3). In addition, we used formulas (Shanock et al., 2010) to perform significance tests for these values.

#### Mediation Test Using the Block Variable Approach

To test Hypothesis 4, we examined the indirect effects of neuroticism congruence/incongruence on the GWB via PR. First, we created a block variable by a weighted linear composite of estimate coefficients (Edwards and Cable, 2009). The block variable represented the joint effect (i.e., congruence and incongruence effect) of the five terms. In the current study, we have hypothesized a positive effect of neuroticism incongruence on older adults’ GWB (i.e., Hypothesis 1). Thus, higher scores of block variable represented higher neuroticism incongruence effect, while lower scores of block variable represented higher neuroticism congruence effect. It is important to note that using the block variable does not change the estimated coefficients for other variables in the equation or the total explained variance (Heise, 1972; Igra, 1979). We regressed PR on the neuroticism block to obtain a regression coefficient referred to as the “a” path. We then regressed GWB on PR (i.e., the “b” path) and the neuroticism block (i.e., the “c” path). In addition, we tested the significance of indirect effects by bootstrapping 1,000 samples.

## Results

### Subject Characteristics

Table 1 shows the characteristics, means and standard deviations of the study variables of the participants. A total of 161 older adults and 72 primary caregivers were included. Of the older adults, 55.28% were women and 56.52% were aged 80 and over. The majority of primary caregivers were also women (90.28%), and the majority were aged 45–59 (68.06%). The results indicated that the GWB of older adults was higher than the Chinese norms (*p* < 0.01) (Wang et al., 1999). The general level of PR was found to be slightly higher than Chinese norms (Cheng et al., 2014). The mean standard *T* scores of neuroticism for older adults and primary caregivers were both classified as an intermediate type (43.3 ∼ 56.7) (Li et al., 2017). In addition, a significant difference in older adults’ GWB (*p* < 0.01) was demonstrated when divided by the PR median (Median = 27), similar to neuroticism in older adults (Median = 41.14).

**TABLE 1:** Participants characteristics, means, and standard deviations of study variables.

	**Older adults (*n* = 161)**		**Primary caregivers (*n* = 72)**		
Gender (*n*, %)	Men	72 (44.72%)	Gender (*n*, %)	Men	7 (9.72%)
	Women	89 (55.28%)		Women	65 (90.28%)
Age (*n*, %)	60–69	30 (18.63%)	Age (*n*, %)	<50	15 (20.83%)
	70–79	40 (24.84%)		50–59	49 (68.06%)
	≥80	91 (56.52%)		≥60	8 (11.11%)
Neuroticism (M ± SD)	44.31 ± 8.46				45.89 ± 8.34
PR (M ± SD)	2.65 ± 0.70				
GWB (M ± SD)	87.35 ± 12.94				

*PR, psychological resilience; GWB, general well-being.*

Table 2 presents an overview of dyads that resulted in congruence and incongruence. Our division criteria was as follows: when older adult’s neuroticism was higher (or lower) than half standard deviation of their primary caregiver’s neuroticism, the dyad was defined incongruent dyad, if not, the dyad was defined congruent dyad (Fleenor et al., 1996). Specifically, 31.06% of the dyads were neuroticism congruence, and 24.84% of the dyads were neuroticism incongruence, in which older adult’s neuroticism was higher than their primary caregiver’s neuroticism. And 44.10% of the dyads were neuroticism incongruence, in which older adults’ neuroticism was lower than that of their primary caregiver.

**TABLE 2:** Descriptive statistics on congruent/incongruent dyads between older adults and primary caregivers (*n* = 161).

**Neuroticism**	**Number of dyads**	**%**
Neuroticism congruence	50	31.06
Neuroticism incongruence (older adults > primary caregivers)	40	24.84
Neuroticism incongruence (older adults < primary caregivers)	71	44.10

### Confirmatory Factor Analysis

As shown in Table 3, we performed CFA to observe the factor structure of the focal variables. Compared to one-factor model and two-factor model, the expected three-factor model (older adults’ neuroticism, older adults’ PR, and older adults’ GWB) displayed a better fit. Compared to other models, RMSEA, NNFI, and CFI of the three-factor model (older adults’ neuroticism, older adults’ PR, and older adults’ GWB) displayed a better fit. In this study, χ^2^/df = 2.49 ≤ 3.0, CFI = 0.91 ≥ 0.9, thus the model was acceptable (Bentler, 1990). In view of the better explanatory power of the three-factor model, we opted to use the three-factor model for further analyses. In the future research, we would screen and include other potential variables and increase the sample size to make the model fit better. Therefore, we concluded that the three variables represented empirically distinct constructs.

**TABLE 3:** Model fit results for confirmatory factor analyses (*n* = 161).

**Model**	**χ^2^**	**df**	**RMSEA**	**CFI**	**TLI**	**Δχ^2^**
M1 (O; PR; GWB)	102.29	41	0.09	0.91	0.87	–
M2 (O+GWB; PR)	132.23	43	0.11	0.87	0.83	29.94
M3 (O+PR; GWB)	183.48	43	0.14	0.79	0.73	81.19
M4 (PR+GWB; O)	178.41	43	0.14	0.8	0.75	76.12
M5 (O+PR+GWB)	208.51	44	0.15	0.75	0.7	106.22

*All alternative models are compared to the hypothesized model (M1). All χ^2^ are significant at *p* < 0.01; O, older adults’ neuroticism; PR, older adults’ psychological resilience; GWB, older adults’ general well-being.*

### Hypothesis Testing

**FIGURE 1:**
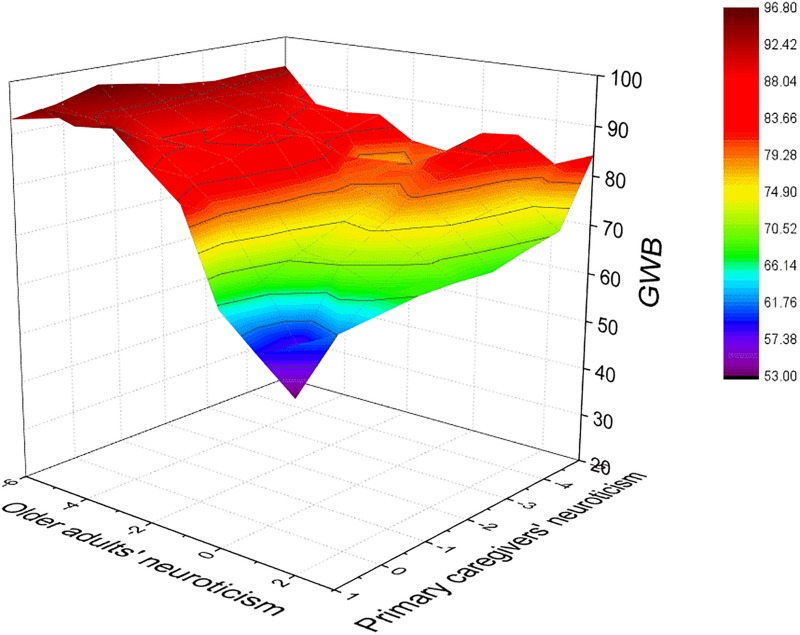
Response surface graph depicting the neuroticism congruence/incongruence effects between older adults and primary caregivers on GWB.

In this study, the average number of older adult for each primary caregiver was 2.2. A correlation analysis was used (Table 4). First, the results indicated that neuroticism was negatively related to PR (*r* = −0.39, *p* < 0.01) and GWB (*r* = −0.61, *p* < 0.01) for older adults. PR was positively related to GWB (*r* = 0.52, *p* < 0.01). Next, to examine the congruence and asymmetrical incongruence effects (Hypotheses 1–3), we performed cross-level polynomial regression and response surface analysis. As shown in Table 5, compared to model 1, the significant increase in *R*^2^ in model 2 presented a non-linear relationship between neuroticism fit and GWB (*△R*^2^ = 0.04, *p* < 0.05). That is to say, compared to older adults’ neuroticism and primary caregivers’ neuroticism, the neuroticism fit between older adults and primary caregivers explained more variation of older adults’ GWB. In Figure 1, the incongruence lines (O = −P) are from the left corner to the right corner, and the congruence lines (O = P) are from the rear corner to the front corner.

**TABLE 4:** Correlation coefficients of variables (*n* = 161).

	**1**	**2**	**3**
1. Older adults’ neuroticism			
2. Primary caregivers’ neuroticism	−0.02		
3. PR	−0.39^∗∗^	0.08	
4. GWB	−0.61^∗∗^	0.01	0.52^∗∗^

**n* = 161 for older adults and *n* = 72 for primary caregivers. PR, psychological resilience; GWB, general well-being. ^∗∗^*p* < 0.01 (two-tailed tests).*

**TABLE 5:** Cross-level polynomial regressions of PR and GWB on neuroticism congruence/incongruence (*n* = 161).

**Variables**	**GWB**	
	
	**Model 1 (*SE*)**	**Model 2 (*SE*)**
Constant	83.49(1.71)	83.74(1.71)
Gender	4.76(1.77)	4.71(1.76)
Age	1.72(1.36)	1.48(1.36)
Primary caregivers’ neuroticism (P)	0.18(0.40)	0.82(0.65)
Older adults’ neuroticism (O)	−2.94(0.39)	−2.84(0.49)
P^2^		0.17(0.14)
O^∗^P		−0.28(0.16)
O^2^		0.05(0.12)
*R*^2^	0.33	0.37
*ΔR^2^*		0.04^∗^
**Congruence (O = P) line**		
Slope a1		−2.02^∗∗^(0.67)
Curvature a2		−0.06(0.22)
**Incongruence (O = −P) line**		
Slope a3		3.65^∗∗^(0.72)
Curvature a4		0.49^∗^(0.20)

*Unstandardized regression coefficients are reported. SE, standard error; PR, psychological resilience; GWB, general well-being; O, older adults’ neuroticism; P, primary caregivers’ neuroticism ^∗^*p* < 0.05 and ^∗∗^*p* < 0.01 (two-tailed tests).*

Hypothesis 1 suggested that neuroticism incongruence between older adults and primary caregivers would predict higher GWB. In this study, the neuroticism incongruence effect of both parties on older adults’ GWB (Hypothesis 1) was tested. Specifically, the curvature along the O = −P line for the surface was significantly positive (a4 = 0.49, *SE* = 0.20, *p* < 0.01) (see Table 5), represented as a U-shape along the O = −P line in the response surface (Figure 1). The convex curvature along the O = −P line indicated that older adults’ GWB was higher when older adults’ neuroticism was incongruent with their primary caregivers’ neuroticism, and any deviation from the O = P line (moving to its left or right) increased older adults’ GWB, thereby supporting Hypothesis 1.

Hypothesis 2 predicted that older adults’ GWB was better when their neuroticism was lower than that of their primary caregivers rather than vice versa. Table 5 shows that the slope of the O = −P line was significant and positive (a3 = 3.65, *SE* = 0.72, *p* < 0.01). Thus, when older adults’ neuroticism was higher than that of their primary caregivers, the older adults’ GWB increased less sharply than it did in the opposite situation. The asymmetrical effect could also be found in Figure 1, in which GWB was higher at the left corner than at the right corner. Hence, Hypothesis 2 was supported.

Hypothesis 3 suggested that older adults’ GWB was higher when both the neuroticism of older adults and primary caregivers were at lower levels. As shown in Table 5, the slope of the O = P line was significant and negative (a1 = −2.02, *SE* = 0.67, *p* < 0.01), indicating that low-low congruence condition predicted higher older adults’ GWB than the high-high congruence condition. After further checking the response surface, we found that GWB at the rear corner was higher than at the front corner. Hence, Hypothesis 3 was verified.

Hypothesis 4 suggested that PR partially mediated the relationship between neuroticism incongruence and older adults’ GWB. Table 6 shows that the neuroticism block was significantly related to PR (path a = 0.39, *SE* = 0.07, *p* < 0.01). Additionally, PR was positively associated with GWB (path b = 0.34, *SE* = 0.07, *p* < 0.01). The effects of the neuroticism block on GWB were also significant (path c’ = 0.46, *SE* = 0.06, *p* < 0.01). The indirect effects of the neuroticism block via PR were significant for GWB [a × b = 0.13, *SE* = 0.04, *p* < 0.01; 95%CI (0.06, 0.20)]. These findings suggest that as the neuroticism incongruence between older adults and primary caregivers increase, older adults’ PR also increases with a corresponding increase in older adults’ GWB. Therefore, PR partially mediated the relationship between the neuroticism incongruence and older adults’ GWB, supporting Hypothesis 4.

**TABLE 6:** Direct, indirect, and total effects of neuroticism block and PR on GWB (*n* = 161).

**Dependent variable**	**PR (*SE*)**	**GWB (*SE*)**
Direct effect of neuroticism block (a path)	0.39^∗∗^ (0.07)	
Direct effect of PR (b path)		0.34^∗∗^ (0.07)
Direct effect of neuroticism block (c’ path)		0.46^∗∗^ (0.06)
Indirect effect of neuroticism block (a × b)		0.13^∗∗^ (0.04)
95% bootstrapped confidence intervals for the indirect effect		0.07, 0.21

*Standardized regression coefficients are reported. SE, standard error; PR, psychological resilience; GWB, general well-being. ^∗∗^*p* < 0.01 (two-tailed tests).*

## Discussion

In this study, using dominance complementarity theory, we examined the interpersonal relationship of neuroticism between older adults and their primary caregivers and further explored its effect on GWB. The results showed that incongruence of neuroticism between older adults and primary caregivers predicted high GWB. In addition, older adults possessed higher GWB when their neuroticism was lower than that of their primary caregiver for older adults in nursing homes, most of their social support comes from their primary caregivers. Previous studies on personality have identified a negative correlation between neuroticism and GWB in older adults (Otonari et al., 2012). This primary result, compared to a personal perspective, contributes to another perspective in predicting older adults’ quality of life in nursing homes.

### Theoretical Implications

The main results of this study have several important theoretical implications. First, we examined neuroticism incongruence between two parties and its effect on GWB (i.e., Hypothesis 1). The results indicated that complementary neuroticism was more likely to improve older adults’ GWB. This finding is similar to a prior study that showed that neuroticism incongruence was beneficial for roommates and that there were more positive emotions and behavior among dissimilar neurotic roommates than similar ones (Luther and Benkenstein, 2017). However, this result is contrary to a longitudinal study of romantic couples that found that moderate congruence in neuroticism predicted higher levels of relationship satisfaction (Hudson and Fraley, 2014). Specifically, a male partner had lower relationship satisfaction in a relationship with dissimilar levels of neuroticism (Hudson and Fraley, 2014). These inconsistent results could be explained as follows. First, different subjects undertake different roles and possess different expectations. Compared to married couples or roommates, older adults and primary caregivers mainly involves taking care, and being taken care of. Their interpersonal interaction focuses more on older adults, when primary caregivers undertake the task of caring for older adults. Hence, the complementarity of neuroticism between these dyads may reduce disharmony, promote relationship satisfaction and increase older adults’ GWB. Moreover, the length of time of interpersonal interaction might be an important influencing factor for GWB (Weidmann et al., 2017). This study selected at least 1 month as the inclusion criteria for primary caregivers, whereas other studies have defined different lengths of time.

Next, we analyzed the incongruence effects on GWB in detail (i.e., Hypothesis 2). The results indicated that older adults showed higher GWB when their neuroticism was lower than that of their primary caregivers. This finding is similar to previous studies. In a person-city personality fit study, Zhou et al. (2017) found that entrepreneurs who were low in neuroticism and conducted their business in a city with a high level of neuroticism enjoyed greater entrepreneurial success. Boele et al. (2017) found that adolescents with higher neuroticism than the classroom norm were at risk of peer victimization. Nevertheless, researches on neuroticism and GWB have confirmed their negative relationship only from personal perspective (Custers et al., 2010; Friedman et al., 2010). It’s worth noting that our results reflected the relative level of neuroticism between dyads (after comparison), rather than the absolute level, that affected older adults’ GWB. To explain Hypothesis 2, we took the characteristic of neuroticism into account. There are several potential reasons for these asymmetric incongruence effects. First, it has been acknowledged that individuals with low levels of neuroticism tend to be in dominant positions, who are more likely to be proactive and to obtain support (Trapnell and Wiggins, 1990; Gilbert and Allen, 1994). When older adult’ neuroticism is lower than that of his/her primary caregiver, the former could obtain more physical and psychological support from the latter, which contributes to the former’s GWB. Second, the root cause of neuroticism is the tendency to generate negative thoughts and emotions on themselves. People with higher neuroticism are more creative in solving problems than those with lower scores, because they tend to pay more attention to problems than those with lower scores (Perkins et al., 2015). Hence, primary caregivers with higher neuroticism might provide more considerate services for older adults and solve problems as soon as possible. Additionally, in this study, according to the scores of neuroticism subscale, we found most of primary caregivers were in middle and stable mood types, and only 10% of them were in unstable types. That is to say, the neuroticism level (mainly at middle and low level) of primary caregivers was higher than that of older adults, which was beneficial to older adults’ GWB. Third, in this study, most of the older adults are over 80 years old. Current study shows that neurotic behavior is controlled by genetic factors, and the genetic differences in neuroticism would be more obvious with the increase of individual age (Eaves and Eysenck, 1976). Thus, the age of older adults may be a potential factor for their GWB, which might be the third reason why the results in this study differs from others. In contrast, when older adults’ neuroticism is higher than that of their primary caregivers, the former would obtain less support and help from their primary caregivers than the opposite situation. And this effect might offset the benefits that complementary neuroticism brings to older adults. Consistent with previous studies, in the case of congruence, older adults’ GWB is higher when the neuroticism of both older adults and their primary caregivers are at lower levels (Zhou et al., 2017).

In particular, researchers have identified PR as a partial mediator of the relationship between neuroticism and happiness and a full mediator of the relationship between neuroticism and positive affect in college students (Lü et al., 2014). Thus, to further investigate this inherent mechanism, we performed mediation analysis to explore the effect of PR on neuroticism fit and GWB. Consistent with prior studies, we found that PR could partially mediate the relationship between neuroticism congruence/incongruence and GWB. Thus, PR could partially interpret the mechanisms of the relationship between neuroticism fit and GWB.

### Practical Implications

Our results suggest that if the neuroticism of primary caregivers is high, this may not be detrimental to the improvement of older adults’ GWB. It is necessary to consider whether the neuroticism fit between the two sides is complementary. Previous studies have shown that a high level of neuroticism can promote people’s creative ability through their higher attention to neuroticism and therefore can promote better problem solving (Perkins et al., 2015). Therefore, it is very important for primary caregivers to pay attention to neuroticism levels to avoid an improvement of the GWB of older adults due to a neuroticism misfit between the two sides. Nursing homes should also pay attention to the suitability of the neuroticism fit between both parties and consider the ability of primary caregivers with different levels of neuroticism because a neuroticism misfit between the two parties could hinder their relationship and the primary caregivers’ capacity development.

Our findings also showed that a similar level of neuroticism between older adults and their primary caregivers was not beneficial for older adults’ GWB. In fact, we found that among the four types of neuroticism fit, older adults’ GWB was higher when the neuroticism fit between both parties was incongruent rather than congruent. In particular, high-level GWB for older adults was found in the neuroticism-incongruent mode for low-level older adults and high-level caregivers. Therefore, nursing homes could produce a decline in the GWB of older adults due to neuroticism incongruence between older adults and their primary caregivers. It is important to ensure a high-low match between the neuroticism levels of both parties.

In nursing homes, primary caregivers usually do not have the opportunity to choose older adults who match their level of neuroticism. Instead, they are often assigned to care for one or more older adults. When higher-neuroticism primary caregivers take care of older adults with the same level of neuroticism, it is difficult to establish a friendly relationship between the two parties, which affects the GWB of older adults. This study also found that resilience was a mediator between neuroticism fit and GWB in older adults. Therefore, we recommend that nursing homes improve the PR of older adults through various means and help to improve their GWB. Relevant systems can be established to enable older adults or primary caregivers to optimize the selection of the other party according to their level of neuroticism, thereby promoting the full utilization of the primary caregivers’ ability and improving the GWB of older adults.

### Limitations and Future Directions

This study has some limitations. First, the data were collected in only one city in China, which significantly limited our findings’ generalizability to community practice. Future research should rely on large samples from multicenters to examine the generalizability and applicability of these findings in different situations. Second, this study was a cross-sectional survey, and it was possible to lose sight of the impact of the timeline on older adults’ GWB. Further investigation could be designed with a follow-up survey, which would provide an overall perspective for analyzing the effect of neuroticism incongruence on older adults’ GWB. Because we were limited to clinical practice, we were unable to achieve a one-to-one pairing of older adults and primary caregivers, which might decrease the strength of our argument to some extent. The average number of older adults for each primary caregiver was in relatively low level, which may influence the statistical power of the analysis, thus further research could expand the sample size for more in-depth research. At last, although the three-factor model was better than other models, it was not quite satisfactory.

## Conclusion

Using dominance complementarity theory, this study showed that neuroticism incongruence between older adults and primary caregivers is beneficial to older adults’ GWB and is partially mediated by PR. Importantly, our results offer potential interventions for reducing the adverse effect of neuroticism and promoting older adults’ GWB in nursing homes. These findings could also be prospectively applied to nursing management for personnel assignment in nursing homes.

## Data Availability Statement

The raw data supporting the conclusions of this manuscript will be made available by the authors, without undue reservation, to any qualified researcher.

## Ethics Statement

This study was carried out in accordance with the recommendations of Ethics Committee at the Hebei Medical University with written informed consent from all subjects. All subjects gave written informed consent in accordance with the Declaration of Helsinki. The protocol was approved by the Ethics Committee at the Hebei Medical University.

## Author Contributions

RH and HD contributed to designing, analyzing, and writing the study. RZ participated in the study’s conception and coordination. PL, PZ, and MZ conceived and collected the data. JH designed the study, analyzed the results, and revised the manuscript.

## Conflict of Interest

The authors declare that the research was conducted in the absence of any commercial or financial relationships that could be construed as a potential conflict of interest.

## Abbreviations

GWB: general well-being
PR: psychological resilience
O: older adults’ neuroticism
P: primary caregivers’ neuroticism.
